# Hypoxia restrains the expression of complement component 9 in tumor-associated macrophages promoting non-small cell lung cancer progression

**DOI:** 10.1038/s41420-018-0064-3

**Published:** 2018-06-07

**Authors:** Lei Li, Hong Yang, Yan Li, Xiao-Dong Li, Ting-Ting Zeng, Su-Xia Lin, Ying-Hui Zhu, Xin-Yuan Guan

**Affiliations:** 1 State Key Laboratory of Oncology in South China and Collaborative Innovation Center for Cancer Medicine, Sun Yat-sen University Cancer Center, 510060 Guangzhou, China; 2 Guangdong Esophageal Cancer Research Institute, 510060 Guangzhou, China; 3 Department of Thoracic Oncology, Sun Yat-sen University Cancer Center, Guangzhou, 510060 China; 4 Department of Pathology, Sun Yat-sen University Cancer Center, 510060 Guangzhou, China; 5 Department of Clinical Oncology, The University of Hong Kong, Hong Kong, China

## Abstract

The tumor microenvironment, including stroma cells, signaling molecules, and the extracellular matrix, critically regulates the growth and survival of cancer cells. Dissecting the active molecules in tumor microenvironment may uncover the key factors that can impact cancer progression. Human NSCLC tumor tissue-conditioned medium (TCM) and adjacent nontumor tissue-conditioned medium (NCM) were used to treat two NSCLC cells LSC1 and LAC1, respectively. Cell growth and foci formation assays were applied to assess the effects of TCM and NCM on cancer cells. The active factors were identified by protein mass spectrometry. Cell growth and foci formation assays showed that 8 of 26 NCM and none of TCM could effectively lead to tumor cell lysis, which was known as tumoricidal activity. And then protein mass spectrometry analysis and functional verifications confirmed that complement component 9 (C9) played a crucial role in the complement-dependent cytotoxicity (CDC)-mediated tumoricidal activity in vitro. Furthermore, immunofluorescent staining revealed that C9 specifically expressed in most alveolar macrophages (AMs) in adjacent lung tissues and a small fraction of tumor-associated macrophages (TAMs) in NSCLC tissues. Most importantly, the percentage of C9-positive cells in AMs or TAMs was responsible for the tumoricidal activity of NCM and TCM. Herein, we found that high expression of C9 in TAMs was a significant independent prognostic factor (*P* = 0.029), and associated with beneficial overall survival (*P* = 0.012) and disease-free survival (*P* = 0.016) for patients with NSCLC. Finally, we unveiled that hypoxic tumor microenvironment could switch the phenotype of macrophages from M1 to M2 forms, accompanying with the downregulation of C9 in TAMs. Collectively, our findings elucidated a novel role of TAMs expressing C9 in the prognosis of NSCLC patients, which provided a promising strategy in the development of anticancer treatments based on the CDC-mediated tumoricidal activity.

## Introduction

Lung cancer is the most common cause of cancer-related death, and generally related to smoking, air pollution, or heredity^1–3^. Non-small cell lung cancer (NSCLC), the major histologic form, accounts for ~80% of all cases, with a 5-year survival rate of only 10–15% for patients in advanced stage^4^. The interactions between tumor cells and immune cells in the tumor microenvironment play an important role in the tumor development^5^. A variety of soluble immune molecules, such as antibodies, cytokines, and complement components, regulate tumor cell survival and metastasis^6,7^.

Innate immunity prevents tumors from developing, and thus exerts a crucial protective role against cancer^8^. Innate immune cells, especially natural killer (NK) cells and macrophages, are attractive effectors as part of an immunotherapeutic strategy. NK cells, unlike the B and T cells of adaptive immunity, are capable of spontaneously destroying cancer cells without prior sensitization^9^. However, most solid tumors are heavily infiltrated with immunosuppressive M2 macrophages that promote tumor growth and metastasis and phenotype and function distinct from activated effector M1 macrophages that have a tumor-destroying role^10^. Many clinically effective tumor-specific mAbs induce direct tumor cell destruction involving innate immune mechanisms; some work via antibody-dependent cell-mediated cytotoxicity, while most involve innate effector cells via complement-dependent cytotoxicity (CDC)^10^.

The complement system, recognized as a main element of the innate immunity, is generally activated by three pathways: classical pathway, alternative pathway, and lectin pathway, participating in three overarching physiologic activities: defending against infection, bridging innate and adaptive immunity, and cleaning apoptotic cells or other waste^11^. Previous studies showed that complement components also played a crucial role in tumor immune surveillance via classical pathway^12^. Initiation of the classical complement pathway occurs when C1, the first complement component, interacts with IgG or IgM antibodies that have already attached to the antigens on the tumor cells^13^. After a cascade of proteolysis, activated C5b bonds to C6–9 that will form a pore in the lipid bilayer membrane of tumor cells, named as membrane attack complex (MAC), which can destroy membrane integrity and proton gradient, lead to cell lysis in a process known as CDC^14,15^. Hence, a mass of MAC deposited on tumor tissues was observed at many kinds of cancer, such as breast cancer^16^ and thyroid carcinoma^17^, which suggested that neoplastic transformation was a potential target of complement system^18^.

Complement component 9 (C9), a terminal component of the complement pathway, was generally produced by the liver and circulated in the plasma. Increased C9 level has been reported in sera samples of patients with squamous cell lung cancer^19^, gastric cancer^20^, and colon cancer^21^, whereas another group has detected upregulation of C9 gene expression in esophageal adenocarcinoma^22^. Therefore, it seems logical that C9 as a biomarker could be used to monitor carcinogenesis and cancer progression, but its precise role in tumor microenvironment is not clear.

## Results

### A tumoricidal microenvironment in NSCLC adjacent nontumor tissue

**Fig. 1:**
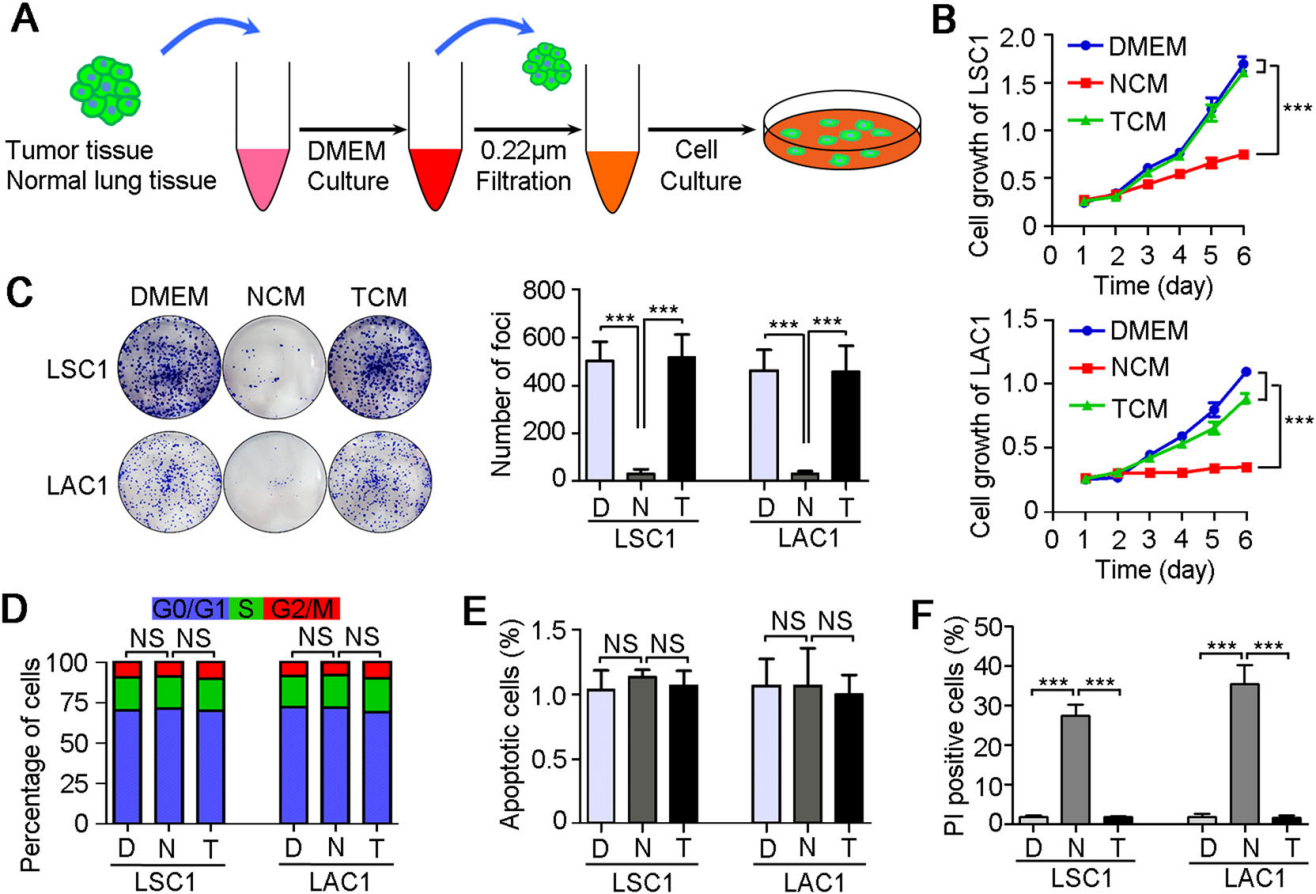
A tumoricidal microenvironment in NSCLC adjacent nontumor tissues. **a** Diagram of tissue culture model. In brief, fresh human NSCLC tumor and paired adjacent nontumor tissues were washed with PBS, and then cultured with DMEM at 37 °C for 2 h. Took out the tissues, and the remaining tissue medium was filtered with 0.22 μm filters and treated for two NSCLC cells, LSC1 and LAC1. Representative results of cell growth tests (**b**), foci formation assays (**c**), cell cycle analyses (**d**), TUNEL apoptosis assay (**e**), and propidium iodide (PI) uptake assay (**f**) of LSC1 and LAC1 cells under the DMEM (D), NCM (N), and TCM (T) treatments. Unsupplemented DMEM was used as a control. All tissue-conditioned media were obtained from one NSCLC patient (Case 4). In all panels, error bars represent SEM. NS no significant difference. ***P < 0.001

In order to understand the reciprocal interactions between tumor cells and soluble factors in tumor microenvironment, 26 pairs of fresh NSCLC and their corresponding adjacent nontumor tissues were collected to prepare the tumor tissue-conditioned media (TCM) and nontumor tissue-conditioned media (NCM), respectively. The NSCLC cell lines (LSC1 and LAC1) were treated with TCM and NCM, respectively (Fig. 1a). Interestingly, 8 of 26 NCM had tumor inhibitory properties in vitro, but neither TCM nor DMEM had any antitumor effects (Table 1 and Fig. 1b, c). The growth curves showed that NCM treatment effectively inhibit the growth of LSC1 and LAC1 cells compared with TCM or DMEM treatments (*P* < 0.001, Fig. 1b). The frequency of foci formation was reduced significantly under the NCM treatment than TCM or DMEM groups (*P* < 0.001, Fig. 1c).

**Table 1:** Clinical samples applied to tissue culture

ID	Gender	Age (years)	Smoking history	Histological subtype	TNM stage	Tumoricidal effect	
						Tumor	Peritumor
1	M	55	+	SCC	IB	−	−
2	M	52	+	ADC	IIA	−	−
3	M	59	+	SCC	IIA	−	−
4	F	30	−	SCC	III	−	+
5	M	69	−	ADC	IIIA	−	−
6	M	66	+	SCC	IB	−	+
7	M	63	−	ADC	IIA	−	−
8	F	51	−	ADC	IIIA	−	−
9	M	32	+	ADC	IIIA	−	−
10	M	54	+	ADC	IIIA	−	+
11	M	71	−	ADC	III	−	−
12	M	60	+	SCC	IIA	−	+
13	M	54	+	SCC	IB	−	+
14	M	71	+	SCC	IIIA	−	−
15	M	73	+	SCC	IIIA	−	−
16	M	49	−	SCC	IIA	−	−
17	M	64	+	SCC	IB	−	−
18	M	70	−	ADC	IA	−	+
19	M	54	+	SCC	IIA	−	−
20	M	56	+	SCC	IIIA	−	−
21	M	38	−	ADC	IIIA	−	−
22	M	71	+	ADC	IB	−	+
23	M	64	+	SCC	IIIA	−	−
24	M	57	+	ADC	IIIA	−	−
25	F	39	+	SCC	IIIA	−	+
26	M	69	+	SCC	IIIB	−	−

*SCC* squamous cell carcinoma, *ADC* adenocarcinoma

To further explore the mechanisms of the antitumor effects of NCM, we analyzed the cell cycle distributions and apoptotic index of LSC1 and LAC1 cells after 3 days of tissue-conditioned media treatments. No significant difference was found between the three treatments (DMEM, NCM, and TCM), which suggested that NCM did not induce tumor cell cycle arrest and apoptosis (Fig. 1d, e). However, propidium iodide uptake assay using fluorescence microscope indicated an increase in plasma membrane permeability and loss of plasma membrane integrity in LSC1 and LAC1 cells treated with NCM, compared with TCM and DMEM treatments (Fig. 1f). These results indicated that tumor cell lysis may be involved in this tumoricidal activity.

### C9 played a crucial role in CDC-mediated tumoricidal activity

**Fig. 2:**
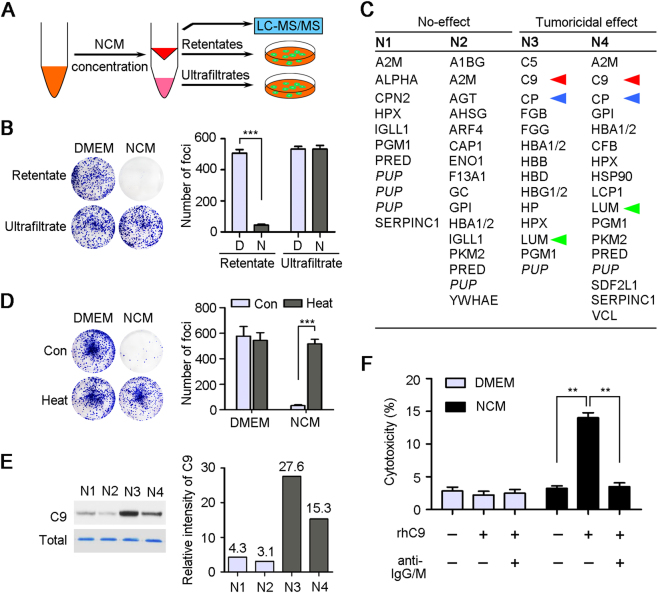
C9 leads to cancer cell lysis in vitro. **a** Diagram of LC-MS/MS analysis of NCM for finding the factors with tumoricidal activity. **b** Representative results of foci formation assay of LSC1 cells under the retentates or ultrafiltrates (Case 10) treatments. D, DMEM; N, NCM. **c** Four NCM samples with (N3, N4, Case 4, and 10) or without (N1, N2, Case 2, and 3) tumoricidal activity were analyzed by LC-MS/MS, and the identified proteins with significant difference between two groups were listed (Supplementary data sheet). Three proteins specifically contained in N3 and N4 group were indicated by red, blue, or green triangles, respectively. PUP putative uncharacterized protein. **d** Representative results of foci formation assays of LSC1 cells under the pre- and post-teat treatments (56 °C water bath for 30 min). **e** The protein levels of C9 in four NCM samples were confirmed by western blot. Total proteins were stained with Coomassie Brilliant Blue as a loading control, and relative expressions of C9 were also quantified. The quantitative values were showed above the bars. **f** Tests of LDH released from LSC1 cells under the DMEM and NCM treatments (Case 10) supplemented with rhC9 (20 μg/ml) and blocking anti-IgG/M (1 μg/ml per antibody), respectively. In all panels, error bars represent SEM. **P *<* 0.01

To further identify the key factors that could lead to tumor cell lysis, four NCM samples with (N3 and N4) or without (N1 and N2) tumoricidal activity were performed protein mass spectrometry analysis. For meeting the requirements of mass spectrometry, the NCM samples were concentrated by ultrafiltration (3-kDa centrifugal filters, Millipore). To confirm the tumoricidal factors deposited in the concentrates that would be analyzed, the retentates and ultrafiltrates were used to treat LSC1 and LAC1 cells again, respectively (Fig. 2a). The representative result of foci formation assay for one sample (N3, Case 10) showed that only the retentate kept the tumoricidal activity, but not the ultrafiltrate (*P* < 0.001, Fig. 2b). Unsurprisingly, a number of soluble factors were identified in the four NCM concentrate samples by protein mass spectrometry, respectively (Fig. 2c and Supplementary data sheet). And three proteins, complement component 9 (C9), ceruloplasmin and lumican, specifically resided in the two samples (N3, and N4) that retained the tumoricidal activity, but not other two samples (N1 and N2) with no effect (Fig. 2c).

Complement-dependent tumor cell lysis is an important way of eliminating tumor^23^. To confirm what we found was complement-mediated cell killing, DMEM and NCM were pretreated with 56 °C water bath for 30 min before cell culture for complete heat inactivation of the complement system^24^. Foci formation assay for one sample (Case 10) showed that the tumoricidal activity of NCM was lost (*P* < 0.001) after heat treatment, but the numbers of foci in DMEM group had no significant difference between pre- and post-heat treatments (Fig. 2d). Furthermore, it has been showed that upregulation of plasma C9 protein was associated with squamous cell lung cancer^19^, gastric cancer^20^, and breast cancer^21^, and our western blot analysis also confirmed that the protein levels of C9 in N3 and N4 was much higher than in N1 and N2 (Fig. 2e). This evidences suggest that C9 may be a key factor in NCM performing tumoricidal activity.

For further verification, recombinant human C9 (rhC9) protein (20 μg/ml, Merck) was supplemented into one NCM sample that had no tumoricidal activity, and the LDH, an indicator of cytotoxicity released into culture medium, was tested after 3 days of treatment. Results showed that the cytotoxicity of NCM group was significantly increased after adding rhC9 protein (*P* < 0.01). However, there was no significant difference between pre- and post-adding rhC9 for DMEM control (Fig. 2f). However, when supplementing human IgG- and IgM-blocking antibodies (1 μg/ml per antibody, Bioss, China) to interfere the initiation of CDC-mediated lysis, the increase of tumoricidal activity of NCM was inhibited dramatically (*P* *<* 0.01), which suggested that the level of C9 was a crucial limit for CDC-mediated tumoricidal effect in vitro (Fig. 2f).

### Alveolar macrophages (AMs) produced C9

**Fig. 3:**
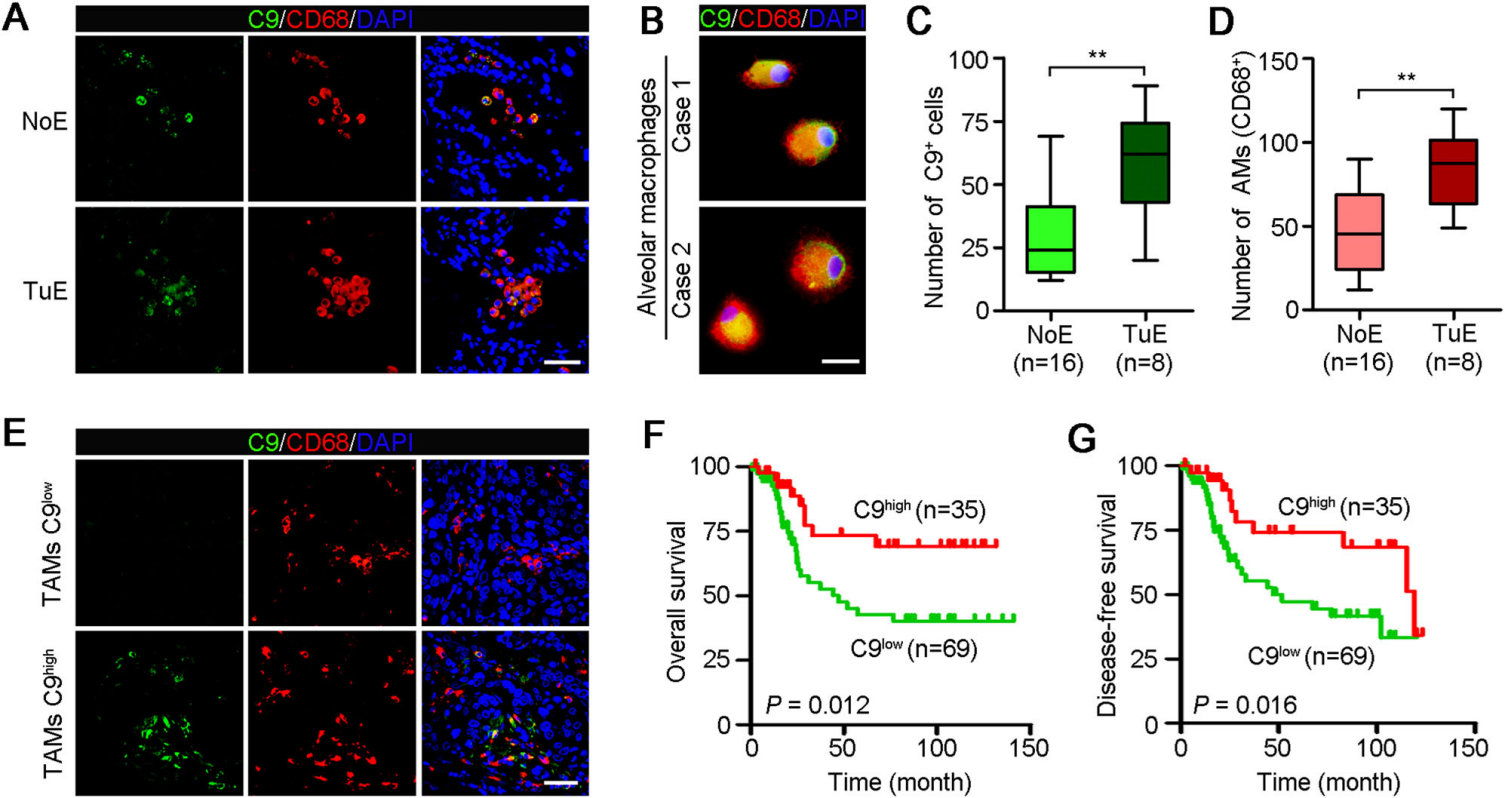
TAMs high expression of C9 associates with superior survival for NSCLC patients. **a** Representative images of two NSCLC adjacent nontumor tissues with no effect (NoE) or tumoricidal effect (TuE) double-stained with antibodies against C9 (green) and CD68 (red). All cells were counterstained with DAPI (blue). Scale bar, 100 μm. **b** Representative images of the co-staining of C9 and macrophage marker CD68 in cultured AMs generated from two NSCLC patients. Scale bar, 10 μm. **c** Numbers of C9-positive cells in adjacent nontumor tissues with or without tumoricidal effect (NoE, *n* = 16; TuE, *n* = 8). Error bars represent SD. **P *<* 0.01. **d** AMs (CD68^+^) numbers between the two groups (NoE, *n* = 16; TuE, *n* = 8). Error bars represent SD. **P *<* 0.01. **e** Representative images of cancer tissues, in which TAMs high (C9^+^/CD68^+^ ≥10%) or low (C9^+^/CD68^+^ <10%) expression of C9, stained with anti-C9 (green) and anti-CD68 (red). All cells were counterstained with DAPI (blue). Scale bar, 100 μm. **f** High expression of C9 in TAMs showed superior overall survival for patients with NSCLC. Log-rank tests, *P* *=* 0.012. **g** TAMs high expression of C9 associates with superior disease-free survival for NSCLC patients. Log-rank tests, *P* *=* 0.016

To identify the source of C9 in the tissue-conditioned medium, immunofluorescence (IF) staining of C9 was performed in 26 human adjacent nontumor tissues with (*n* = 8) or without (*n* = 18) tumoricidal activity. The results showed that C9 was expressed specifically at alveolar space (Fig. 3a). Because AMs are the major cells in human pulmonary alveoli and has innate immune defense function^25^, we co-stained the CD68 (a marker of macrophage) and C9 in lung tissues to investigate if AMs expressed C9. Results showed that only AMs were C9 positive (Fig. 3a). Moreover, we also performed the co-staining of C9 and CD68 in primary AMs generated from two NSCLC patients, which showed that AMs could indeed secrete C9 (Fig. 3b). In addition, nontumor tissues with tumoricidal activity contained more C9-positive cells than others without effect (*P* < 0.01, Fig. 3c), which further supporting our in vitro finding that C9 plays crucial role in tumor cell lysis. More AMs were contained in nontumor tissues with tumoricidal activity than others without effect (*P* < 0.01, Fig. 3d), revealing that the tumoricidal activity of NCM was related to the number of AMs.

### High expression of C9 in TAMs associates with superior survival for NSCLC patients

Our study shows, for the first time, that AMs can secrete C9 (Fig. 3a, b), but the evidences about the expression of C9 in TAMs have not been provided. To determine whether C9 expression in NSCLC TAMs, double IF staining of C9 and CD68 was performed in cancer tissues that had been showed without tumoricidal activity. IF staining showed lower expression of C9 in TAMs (C9^+^/CD68^+^ <40%, Fig. 3e) than that in AMs (C9^+^/CD68^+^ >50%, Fig. 3a). To further investigate the role of TAMs-secreted C9 in NSCLC progression, more cancer tissues (total, *n* = 104) were analyzetd by IF double-staining. In all, 35/104 of cancer tissues showed relative higher expression of C9 in TAMs (C9^+^/CD68^+^ ≥10%) compared with the others (C9^+^/CD68^+^ <10%, Fig. 3e). Next, the correlations between the expression level of C9 in TAMs and the clinicopathological characteristics of NSCLC patients were also evaluated. Results showed that high expression of C9 in TAMs positively associated with tumor necrosis (*P* = 0.024) and was negatively correlated with lymph node metastasis (*P* = 0.025, Table 2), suggesting that C9 played a crucial role in NSCLC development. Moreover, Kaplan–Meier analysis also revealed that low expression of C9 in TAMs significantly associated with worse overall survival (*P* = 0.012, Fig. 3f) and disease-free survival (*P* = 0.016, Fig. 3g). And the multivariate analysis using the Cox proportional hazards model demonstrated that high expression of C9 in TAMs was an independent prognostic factor for patients with NSCLC (*P* = 0.029, Table 3). Summarily, these findings suggested that high-expression C9 in TAMs was a benefit for patient survival.

**Table 2:** Correlation between C9 expression in TAMs and clinicopathological characteristics of NSCLC patients (*n* = 104)

Characteristics	Number	C9 expression		Chi-squared test *P* value
		Low (*n* = 69)	High (*n* = 35)	
Gender				1.000
Male	90	60	30	
Female	14	9	5	
Age (years)				0.651
≤65	73	47	26	
>65	31	22	9	
Smoking history				0.645
Smokers	77	50	27	
Never smokers	27	19	8	
Histological subtype				0.460
SCC	81	52	29	
ACC	23	17	6	
Adjacent organs invasion				0.813
Negative	27	17	10	
Positive	77	52	25	
Tumor size (cm)				0.655
≤5	72	49	23	
>5	32	20	12	
Lymph node metastasis				0.024*
Negative	49	27	22	
Positive	55	42	13	
Necrosis				0.025*
Negative	74	54	20	
Positive	30	15	15	
TNM stage				0.294
I+II	64	40	24	
III+IV	40	29	11	

*AMs* tumor-associated macrophages, *SCC* squamous cell carcinoma, *ACC* adenocarcinoma

*P < 0.05, overall

**Table 3:** Univariate and multivariate analyses of overall survival in NSCLC patients (*n* = 104)

Variable	Univariate analysis			Multivariate analysis		
	Risk ratio	95% CI	*P*	Risk ratio	95% CI	*P*
Gender	0.278	0.257–2.058	0.549			
Male vs. female						
Age (years)	1.029	0.995–1.064	0.090			
≤65 vs. >65						
Smoking history	1.603	0.667–3.853	0.291			
Smokers vs. never smokers						
Histological subtype	1.338	0.518–3.453	0.547			
SCC vs. ACC						
Adjacent organs invasion	0.806	0.402–1.614	0.542			
Negative vs. positive						
Tumor size (cm)	1.017	0.881–1.174	0.821			
≤5 vs. >5						
Necrosis	1.177	0.579–2.393	0.652			
Negative vs. positive						
Lymph node metastasis	3.311	1.665–6.583	0.001*	1.802	0.684–4.746	0.233
Negative vs. positive						
TNM stage	1.941	1.322–2.850	0.001*	1.498	0.867–2.587	0.148
I+II vs. III+IV						
C9 expression of TAMs	0.367	0.167–0.807	0.013*	0.413	0.186–0916	0.029*
High vs. low						

*CI* confidence interval, *TAMs* tumor-associated macrophages, *SCC* squamous cell carcinoma, *ACC* adenocarcinoma

*P < 0.05, overall

### Hypoxia environment inhibited C9 expression in M2 macrophages

**Fig. 4:**
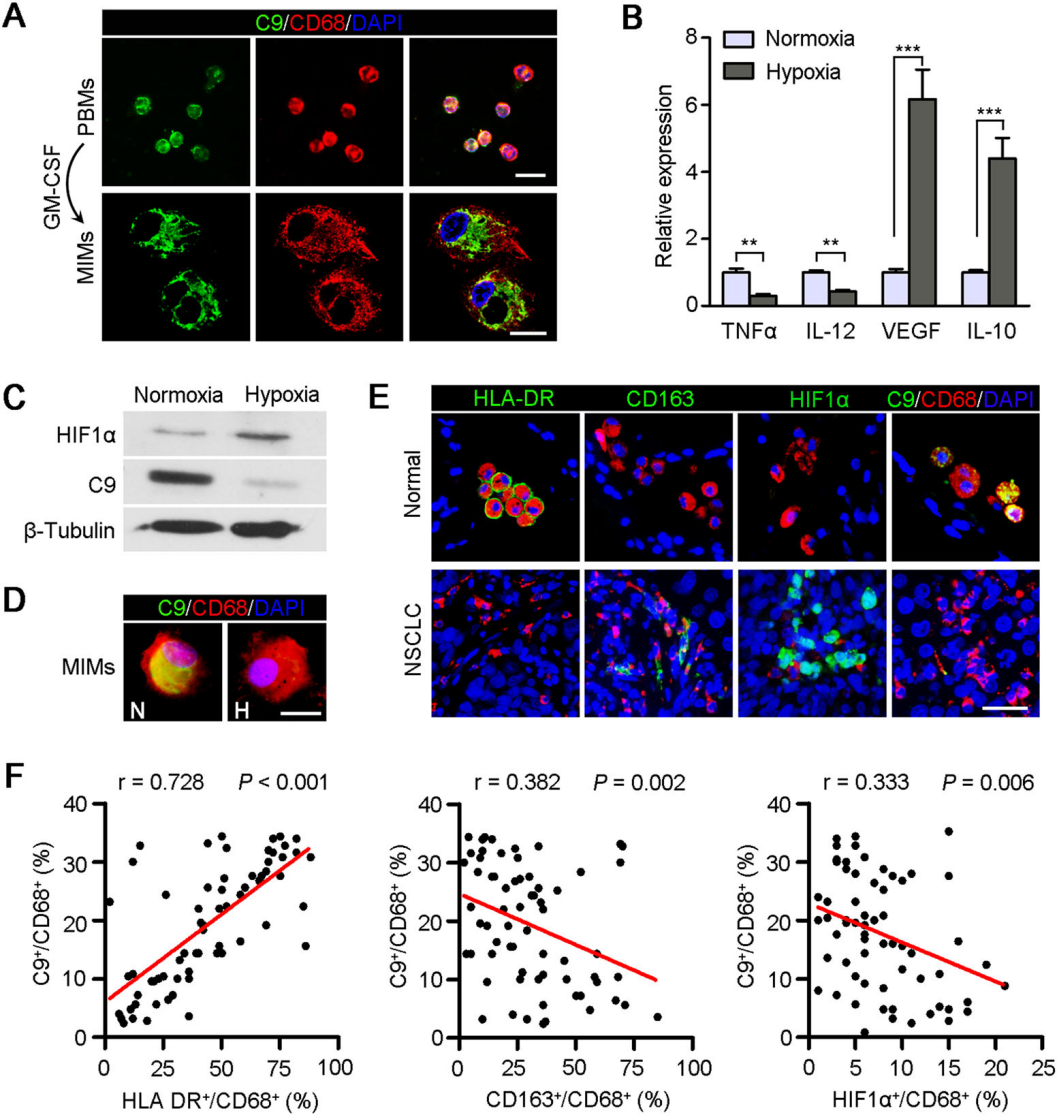
Hypoxia reduces C9 expression in macrophages. **a** Representative images of PBMs and MIMs (induced by GM-CSF, 500 U/ml) stained with anti-C9 (green) and anti-CD68 (red). All cells were counterstained with DAPI (blue). Scale bar, 50 μm in PBMs, scale bar, 10 μm in MIMs. **b** Relative expressions of TNFα, IL-12, VEGF, and IL-10 in MIMs in normoxia and hypoxia environments. **P *<* 0.01. ***P *<* 0.001. **c** Western blot analysis of the expressions of C9 and HIF1α in MIMs in hypoxia environment. β-Tubulin was used as a loading control. **d** Representative images of MIMs in normoxia and hypoxia environments stained with anti-C9 (green) and anti-CD68 (red). All cells were counterstained with DAPI (blue). N, normoxia; H, hypoxia. Scale bar, 10 μm. **e** Representative images of adjacent nontumor and NSCLC tissue sections stained with HLA-DR (green), CD163 (green), HIF1α (green), and C9 (green), in combination with CD68 (all, red), respectively. All cells were counterstained with DAPI (blue). Scale bar, 50 μm. **f** Scatter plots of the proportions of HLA-DR^+^/CD68^+^ (M1 macrophages), CD163^+^/CD68^+^ (M2 macrophages), or HIF1α^+^/CD68^+^ vs. C9^+^/CD68^+^ in 66 NSCLC samples

Both AMs and TAMs are differentiated from peripheral blood monocytes (PBMs)^26,27^. Next, we used monocyte-induced macrophages (MIMs) to dissect the mechanisms of the low expression of C9 in TAMs. First, PBMs were separated from healthy donators and differentiated into macrophages in the presence of the recombinant human granulocyte–macrophage colony-stimulating factor (rhGM-CSF, 500 U/ml) for 2 weeks^28^. IF staining showed that both PBMs and MIMs expressed C9 protein, and the characteristics of macrophage, such as vacuolization, eccentric nucleus, and microvilli formation, were observed with laser confocal microscope (Fig. 4a).

Hypoxic tumor microenvironment is a salient hallmark of solid tumor and modulates tumor development^29^, so we first treated MIMs at low-oxygen levels (1% O_2_) to test the effect of hypoxia on the expression of C9 in macrophages. It is well known that macrophages display plasticity by reversibly transitioning between M1 (with antitumor function) and M2 (with pro-tumor function) states^30^. Herein, hypoxia induced the macrophage forms transition may be responsible for the downregulation of C9. To confirm the hypothesis, we tested the expressions of four representative markers, TNFα and IL-12 high expressed in M1 macrophages, VEGF and IL-10 high expressed in M2 macrophages, by real-time quantification PCR, respectively (Fig. 4b). Results showed that hypoxia could induce the macrophage–phenotype transformation from M1 to M2 forms. Both western blot assay (Fig. 4c) and IF staining (Fig. 4d) indicated that the protein level of C9 was significantly decreased after 12 h hypoxia treatment, compared with normoxia culture, which accounted for the down expression of C9 in TAMs in tumor microenvironment.

In order to further assess whether the expression of C9 was related to the different cellular subsets of macrophages, paired tissue sections from 66 NSCLCs were double-stained for HLA-DR/CD68 (M1 macrophages), CD163/CD68 (M2 macrophages), HIF1α/CD68, and C9/CD68, respectively^31^. In adjacent nontumor tissues, AMs were high-expressed HLA-DR and C9, but not CD163 and HIF1α, which indicates that AMs, belonging to M1 form, performed innate antitumor immunity with C9 in sufficient oxygen environment (Fig. 4e). But in tumor, a lot of TAMs were CD163 and HIF1α-positive, and less expressed HLA-DR and C9 (Fig. 4e). Scatter plots of the proportions of HLA-DR^+^/CD68^+^ (M1 form), CD163^+^/CD68^+^ (M2 form) or HIF1α^+^/CD68^+^ vs. C9^+^/CD68^+^ showed that the C9 expression levels in TAMs were positive correlated with the M1 macrophage densities (*P* < 0.001) and negative correlated with the densities of M2 macrophage (*P* = 0.002) and the percentage of HIF1α-positive macrophage (*P* = 0.006) (Fig. 4f). This data convincingly proved that the expression of C9 in tumor microenvironment was downregulated with the switch of the subsets of macrophages from M1 to M2 forms in hypoxia environment.

## Discussion

Soluble factors, an essential component of tumor microenvironment, regulate tumor development^32^. To study the functions of secretory factors in NSCLC, we used tissue-conditioned medium to treat lung cancer cells in vitro. During a short time of tissue culture with DMEM, a mass of soluble factors deposited in tumor microenvironment will release into tissue medium. Interestingly, we found that only a part of NCM samples, but not TCM, could dramatically inhibit tumor cell growth, compared with DMEM control. These results show two clues: generally speaking, tumor microenvironment contains many growth factors, such as EGF, FGF, and IGF^33^, that can promote cell proliferation, but our in vitro tissue culture experiments show no significant difference between TCM and DMEM groups; importantly, some soluble factors with tumoricidal activity deposit in adjacent nontumor tissues. Therefore, to find this tumoricidal factors in NCM, we performed protein mass spectrometry analysis. And finally, protein C9 was confirmed as a crucial limit in CDC pathway that was responsible for the tumoricidal activity of NCM.

More remarkable, although C9 was found directly from NCM, we could not rule out the possibility of blood contamination, because a few of plasma proteins were also identified in all samples, such as hemoglobin and serum albumin precursor (Supplement data sheet). And, except for C9, many other complement components were also contained in NCM, such as complement C3, C4a, C5, and complement factor B, which also confirmed our findings about CDC in vitro. However, generally, MAC contains up to 22 molecules of C9^34^, far more than other complement components, which suggests that enough C9 is needed for MAC formation in CDC progress. Hence, we next studied the expression of C9 in adjacent nontumor tissues by IF staining, and results showed that AMs specifically expressed C9 protein and the number of C9-positive cells was associated with the tumoricidal activity. Therefore, a conclusion is drawn that resident macrophage-derived C9 can inhibit tumor progression combining with systemic complement components. In other words, local C9 is an important supplement for CDC-mediated immune surveillance in tumor microenvironment.

Furthermore, we find that TAMs also express C9, but the percentage of C9 positive in TAMs is far less than that in AMs. We next investigated the clinicopathologic correlation of C9 expression in TAMs in 104 NSCLC patients. Results showed that high expression of C9 in TAMs associated with necrosis inside of cancer tissues, suggesting that CDC might lead to a large number of tumor cells lysis. However, in general, tumor necrosis was owing to lack angiogenesis for nutrition supply or immune cell attacks, but our findings suggested that complements system could also attack tumor cells in tumor microenvironment. But the expression of C9 in TAMs is not related to the tumor size, which may be because that some tumor cells can escape the complement attack via many mechanisms. For instance, previous studies have showed that tumor cells are high expression of membrane complement regulatory proteins, such as CD46, CD55, and CD59^35,36^, that usually protected host cells from complement-mediated destruction, and secrete many soluble complement inhibitors, such as C1 inhibitor^37^, factor I^38^, and factor H^39^. Moreover, high expression of C9 in TAMs also associates with less lymphatic metastasis. TAMs often infiltrates into tumor stroma, especially around blood vessels and lymphatic vessels^34,40^, which may form an immune barrier for preventing metastasis. Hence, we found that high expression of C9 in TAMs could successfully distinguish a set of patients with superior survival.

We further explored the reasons of downregulation in most TAMs. It is well known that both AMs and TAMs are differentiated from PBMs^27,28^, but the expressions of C9 between them are significantly different despite the high expression of C9 in PBMs, which indicates that the specific tumor microenvironment may regulate the expression of C9 in TAMs. As hypoxia is a main hallmark of tumor microenvironment, we treated the MIMs in low-oxygen environment for 12 h and tested the expression of C9. Our data showed that the expression of MIMs was significantly inhibited in hypoxia treatment. Researches have showed that resident macrophages can be divided into M1- and M2-type subpopulations based on their dual functions of anti- and pro-tumor development, and hypoxia can modify macrophages from a killing capacity (M1 form) to a promoting form (M2 form)^41,42^. And our study also showed that the downregulation of C9 was involved in this phenotype shift of macrophages. Nevertheless, the concrete mechanisms of downregulation of C9 in TAMs in hypoxic environment were needed to further study to more clearly understand the strategy of lung cancer cells eluding CDC.

In conclusion, we first find that AMs were active participators in the suppression of cancer development via secreting C9 and other tumoricidal factors. However, in tumor microenvironment, hypoxia switched the immunophenotype of macrophages from M1 to M2 forms, which accompanying with the downregulation of C9 in TAMs, and leading to loss of tumoricidal activity. Hence, inverting macrophages forms from M2 to M1 to promote secreting C9 in tumor microenvironment may be a promising strategy for suspending the NSCLC progression.

## Materials and methods

### Clinical specimens and cell lines

A total of 104 paired specimens (tumor and adjacent nontumor tissues) were collected from the Sun Yat-sen University Cancer Center (Guangzhou, China) and approved by the Committees for Ethical Review of Research at Sun Yat-sen University. No patients recruited in this study received preoperative chemotherapy or radiotherapy. Human non-small cell lung cancer (NSCLC) cell lines LSC1 (also known as SCC210011) and LAC1 (also known as ADC212102) were established in our laboratory^43^ and cultured in DMEM-complete medium (containing 10% FBS). The cells were incubated at 37 °C in a humidified chamber containing 5% CO_2_.

### Preparation of tissue-conditioned media

The fresh tumor and adjacent nontumor tissue specimens were collected immediately after the surgery resection, and then rinsed thrice with phosphate buffer solution (PBS). All tissues were cut into 5–10 mm^3^ fragments with scalpels and incubated with unsupplemented DMEM for 2 h at 37 °C in a humidified chamber containing 5% CO_2_. The supernatant was then collected and centrifuged at 1000 rpm for 5 min, followed by filtering using a 0.22 μm filter. Subsequently, the tissue-conditioned media was added to cell cultures at a 1:5 ratio with DMEM-complete medium. Unsupplemented DMEM was also diluted with DMEM-complete medium as a control.

### Foci formation assay

Briefly, 1 × 10^3^ cells were seeded in each well of a 6-well plate with tissue-conditioned media or control medium. After a week of culture, surviving colonies (>50 cells per colony) were counted with crystal violet (Sigma) staining. Triplicate-independent experiments were performed.

### Cell proliferation assay

The Cell Counting Kit-8 (CCK-8) assay kit (Dojindo Corp. Japan) was applied to measure the cell growth rate. A suspension of 1 × 10^3^ cells was planted in each well of a 96-well plate, in which 10 μl CCK-8 was added to 90 μl of culture medium. And after 2 h incubation at 37 °C, the absorbance was measured at 450 nm. Triplicate-independent experiments were performed.

### TUNEL apoptosis assay

The apoptotic index was analyzed with the In Situ Cell Death Detection Kit (#11684817910, Roche). In brief, 1 × 10^4^ LSC1 or LAC1 cells were planted on the slide in the 24-well plate, respectively. After 3 days of NCM, TCM, and DMEM treatments, the cells were fixed with 4% paraformaldehyde for 1 h at room temperature. The slide was rinsed three times with PBS, and incubated with blocking solution (3% H_2_O_2_ in methanol) for 10 min at room temperature. Next, tumor cells were incubated in permeabilisation buffer (0.1% Triton X-100 in 0.1% sodium citrate) for 2 min on ice. TUNEL reaction mixture was added on the slide and incubated for 1 h at 37 °C in a humidified atmosphere in the dark. Tumor cells on slide were rinsed three times with PBS and counterstained with DAPI (Life Technology). The apoptotic cells were detected with a confocal laser-scanning microscope (Olympus FV1000) in the range of 515–565 nm.

### Mass spectrometry

The tissue-conditioned media were prepared for LC-MS/MS analyses as followed. Four adjacent nontumor tissue-conditioned media samples, including two with tumoricidal effects and two without effects, were concentrated using a Centricon (Amicon Ultra centrifugal filter, 3-kDa; Millipore, USA). The >3-kDa fraction remained above the filter was then digested by trypsin (Sigma) and analyzed with mass spectrometry, which was conducted as described previously^44,45^.

### Western blot analysis

Western blot analysis was performed according to the standard protocol with antibodies against C9 (Abcam), HIF1α (Cell Signaling Technology) and β-Tubulin (Cell Signaling Technology). Signals were quantified by ImageJ software (http://rsb.info.nih.gov/ij) and defined as the ratio of target protein to total protein that stained with Coomassie Brilliant Blue (Sigma).

### Immunofluorescence staining

For tissue samples, the paraffin-embedded tissue sections were deparaffinized and rehydrated. Slides were boiled in 10 mM citrate buffer (pH 6.0) for 40 min for the antigen retrieval. For cells staining, cells were fixed with 4% paraformaldehyde for 10 min at room temperature, and washed thrice with PBS. Nonspecific binding was blocked with 5% bovine serum albumin for 30 min. The slides were incubated with the primary antibodies against complement component 9 (C9, Abcam), HLA-DR (Abcam), CD163 (Abcam), HIF1α (Cell Signaling Technology), and CD68 (Abcam) at 4 °C overnight, followed by incubation with Alexa Fluor® 594 or 488-conjugated secondary antibodies (Invitrogen). For confocal microscopy, the cells on coverslips were counterstained with DAPI (Life Technology) and imaged using a confocal laser-scanning microscope (Olympus FV1000).

### Cytotoxicity assay

When cell plasma membrane damaged, lactic dehydrogenase (LDH), an indicator of cytotoxicity, was released into the culture medium. The amount of LDH was measured by a LDH Cytotoxicity Assay Kit (Beyotime Biotechnology, Suzhou, China).

### Preparation of macrophages

Peripheral blood monocytes (PBMs) from a healthy donor were isolated by density-gradient centrifugation using Ficoll Paque PLUS (GE Healthcare). For macrophages inducement, PBMs were treated with rhGM-CSF (500 U/ml, Amoytop Biotech, Xiamen, China) for 2 weeks in DMEM-complete medium. The morphological characteristics of monocyte-induced-macrophages (MIMs) were observed with laser confocal microscope (OLYMPUS FV1000, Japan).

### Hypoxia treatment

MIMs were seeded in a culture dish (diameter 6 cm) for overnight. Replacing culture medium, and putting the dish into an incubator that brimming with 1% oxygen for culture 12 h at 37 °C. After hypoxia treatment, washing cells three times with PBS rapidly, and then extracting cell protein or total RNA. For control, same number of MIMs was cultured in a carbon dioxide incubator with normoxia.

### Quantitative polymerase chain reaction (qPCR)

Total RNA was extracted using the Trizol Reagent (Roche) and reverse transcription was performed using the Reverse Transcriptase (Takara). The complementary DNA was subjected to quantitative real-time PCR using the SYBR Green PCR Kit (Roche) and the assay was performed on an ABI PRISM 7900 Sequence Detector. β-Actin was used as an internal control. The primer sequences: TNFα, 5′-TGGGATCATTGCCCTGTGAG-3′ (forward) and 5′-GGTGTCTGAAGGAGGGGGTA-3′ (reverse); IL-12, 5′-TTCGCTTTCATTTTGGGCCG-3′ (forward) and 5′-ATCAGCTTCTCGGTGACACG-3′ (reverse); VEGF, 5′-CTGTCTAATGCCCTGGAGCC-3′ (forward) and 5′-ACGCGAGTCTGTGTTTTTGC-3′ (reverse); IL-10, 5′-TGTTCTTTGGGGAGCCAACA-3′ (forward) and 5′-GGGCTCCCTGGTTTCTCTTC-3′ (reverse); β-Actin, 5′-CATGTACGTTGCTATCCAGGC-3′ (forward) and 5′-CTCCTTAATGTCACGCACGAT-3′ (reverse). The value of relative expression of target genes (2^-ΔΔCt^) was normalized to the endogenous β-actin reference (ΔCt).

### Statistics

Statistical analyses were performed using the SPSS 18.0 (SPSS, Inc., Chicago, USA). The independent Student’s *t* test was used to assess the statistical significance between any two preselected groups. The two-tailed chi-squared test was applied to analyze the association of tumor-associated macrophages (TAMs) C9 expression with different clinicopathological characteristics. Univariate analysis was conducted by log-rank test and the Cox proportional hazards model was used in the multivariate analysis. Survival curves were estimated using the Kaplan–Meier plots. The Spearman’s rank correlation coefficient was calculated to assess the relationships between M1 and M2 macrophage densities, HIF1α expression, and C9 expression in TAMs. A significant difference was considered statistically when *P* value was <0.05.

## Electronic supplementary material

Supplementary Mass Spectrometry Datasheet
