# Supplementary material for: Hypoxia restrains the expression of complement component 9 in tumor-associated macrophages promoting non-small cell lung cancer progression
Source: Cell Death Discov. 2018 Jun 7;4:63. doi: 10.1038/s41420-018-0064-3 (PMC5992192; doi:10.1038/s41420-018-0064-3)
Supplement: Supplementary file 1 — Supplementary Mass Spectrometry Datasheet [file 41420_2018_64_MOESM1_ESM.docx]

**Supplementary datasheet A** Proteins identified by Mass Spectrometry in NCM1

| ID | Description | Accession Number | Mass | Ions score>38, *P*<0.05 | Queries matched | emPAI |
| --- | --- | --- | --- | --- | --- | --- |
| 1 | ALB Putative uncharacterized protein ALB | [IPI00022434](http://shh-b7892952567/mascot/cgi/protein_view.pl?file=../data/20140403/F001631.dat&hit=IPI00022434&px=1&ave_thresh=38&_sigthreshold=0.05&_server_mudpit_switch=0.001) | 73881 | 5542 | 309 | 19.82 |
| 2 | similar to immunoglobulin lambda-like polypeptide 1 | [IPI00936490](http://shh-b7892952567/mascot/cgi/protein_view.pl?file=../data/20140403/F001631.dat&hit=IPI00936490&px=1&ave_thresh=38&_sigthreshold=0.05&_server_mudpit_switch=0.001) | 53960 | 746 | 33 | 0.31 |
| 3 | TF Serotransferrin | [IPI00022463](http://shh-b7892952567/mascot/cgi/protein_view.pl?file=../data/20140403/F001629.dat&hit=IPI00022463&px=1&ave_thresh=38&_sigthreshold=0.05&_server_mudpit_switch=0.001) | 79280 | 639 | 19 | 0.32 |
| 4 | IGHV4-31 Putative uncharacterized protein DKFZp686N02209 | [IPI00384938](http://shh-b7892952567/mascot/cgi/protein_view.pl?file=../data/20140403/F001631.dat&hit=IPI00384938&px=1&ave_thresh=38&_sigthreshold=0.05&_server_mudpit_switch=0.001) | 53503 | 411 | 19 | 0.71 |
| 5 | HPX Hemopexin | [IPI00022488](http://shh-b7892952567/mascot/cgi/protein_view.pl?file=../data/20140403/F001629.dat&hit=IPI00022488&px=1&ave_thresh=38&_sigthreshold=0.05&_server_mudpit_switch=0.001) | 52385 | 263 | 8 | 0.23 |
| 6 | IGHG4 Putative uncharacterized protein IGHG4 (Fragment) | [IPI00830132](http://shh-b7892952567/mascot/cgi/protein_view.pl?file=../data/20140403/F001631.dat&hit=IPI00830132&px=1&ave_thresh=38&_sigthreshold=0.05&_server_mudpit_switch=0.001) | 43856 | 260 | 5 | 0.28 |
| 7 | IGHG2 Putative uncharacterized protein DKFZp686I04196 (Fragment) | [IPI00399007](http://shh-b7892952567/mascot/cgi/protein_view.pl?file=../data/20140403/F001631.dat&hit=IPI00399007&px=1&ave_thresh=38&_sigthreshold=0.05&_server_mudpit_switch=0.001) | 46716 | 221 | 11 | 0.17 |
| 8 | ITIH4 Isoform 2 of Inter-alpha-trypsin inhibitor heavy chain H4 | [IPI00218192](http://shh-b7892952567/mascot/cgi/protein_view.pl?file=../data/20140403/F001631.dat&hit=IPI00218192&px=1&ave_thresh=38&_sigthreshold=0.05&_server_mudpit_switch=0.001) | 101520 | 180 | 6 | 0.11 |
| 9 | SERPINA1 Isoform 1 of Alpha-1-antitrypsin | [IPI00553177](http://shh-b7892952567/mascot/cgi/protein_view.pl?file=../data/20140403/F001629.dat&hit=IPI00553177&px=1&ave_thresh=38&_sigthreshold=0.05&_server_mudpit_switch=0.001) | 46878 | 176 | 18 | 1.00 |
| 10 | Putative uncharacterized protein | [IPI00550731](http://shh-b7892952567/mascot/cgi/protein_view.pl?file=../data/20140403/F001631.dat&hit=IPI00550731&px=1&ave_thresh=38&_sigthreshold=0.05&_server_mudpit_switch=0.001) | 26503 | 135 | 3 | 0.49 |
| 11 | IGHA1 SNC66 protein | [IPI00383164](http://shh-b7892952567/mascot/cgi/protein_view.pl?file=../data/20140403/F001631.dat&hit=IPI00383164&px=1&ave_thresh=38&_sigthreshold=0.05&_server_mudpit_switch=0.001) | 54601 | 111 | 2 | 0.07 |
| 12 | Putative uncharacterized protein | [IPI00550731](http://shh-b7892952567/mascot/cgi/protein_view.pl?file=../data/20140403/F001629.dat&hit=IPI00550731&px=1&ave_thresh=38&_sigthreshold=0.05&_server_mudpit_switch=0.001) | 26503 | 97 | 2 | 0.14 |
| 13 | C3 Complement C3 (Fragment) | [IPI00783987](http://shh-b7892952567/mascot/cgi/protein_view.pl?file=../data/20140403/F001631.dat&hit=IPI00783987&px=1&ave_thresh=38&_sigthreshold=0.05&_server_mudpit_switch=0.001) | 188569 | 89 | 6 | 0.02 |
| 14 | Putative uncharacterized protein DKFZp686C02220 (Fragment) | [IPI00423461](http://shh-b7892952567/mascot/cgi/protein_view.pl?file=../data/20140403/F001629.dat&hit=IPI00423461&px=1&ave_thresh=38&_sigthreshold=0.05&_server_mudpit_switch=0.001) | 55038 | 87 | 1 | 0.07 |
| 15 | A2M Alpha-2-macroglobulin | [IPI00478003](http://shh-b7892952567/mascot/cgi/protein_view.pl?file=../data/20140403/F001629.dat&hit=IPI00478003&px=1&ave_thresh=38&_sigthreshold=0.05&_server_mudpit_switch=0.001) | 164600 | 62 | 2 | 0.02 |
| 16 | highly similar to ALPHA-1-ANTICHYMOTRYPSIN | [IPI00550991](http://shh-b7892952567/mascot/cgi/protein_view.pl?file=../data/20140403/F001629.dat&hit=IPI00550991&px=1&ave_thresh=38&_sigthreshold=0.05&_server_mudpit_switch=0.001) | 50737 | 61 | 1 | 0.07 |
| 17 | PRED58 protein (Fragment) | [REV_IPI00022050](http://shh-b7892952567/mascot/cgi/protein_view.pl?file=../data/20140403/F001631.dat&hit=REV_IPI00022050&px=1&ave_thresh=38&_sigthreshold=0.05&_server_mudpit_switch=0.001) | 26854 | 59 | 6 | 0.14 |
| 18 | PGM1 Isoform 2 of Phosphoglucomutase-1 | [IPI00217872](http://shh-b7892952567/mascot/cgi/protein_view.pl?file=../data/20140403/F001631.dat&hit=IPI00217872&px=1&ave_thresh=38&_sigthreshold=0.05&_server_mudpit_switch=0.001) | 64092 | 50 | 2 | 0.06 |
| 19 | SERPINC1 Antithrombin-III | [IPI00032179](http://shh-b7892952567/mascot/cgi/protein_view.pl?file=../data/20140403/F001631.dat&hit=IPI00032179&px=1&ave_thresh=38&_sigthreshold=0.05&_server_mudpit_switch=0.001) | 53025 | 50 | 4 | 0.07 |
| 20 | CPN2 Carboxypeptidase N subunit 2 | [IPI00479116](http://shh-b7892952567/mascot/cgi/protein_view.pl?file=../data/20140403/F001629.dat&hit=IPI00479116&px=1&ave_thresh=38&_sigthreshold=0.05&_server_mudpit_switch=0.001) | 61431 | 43 | 1 | 0.06 |
| 21 | LTA4H Isoform 2 of Leukotriene A-4 hydrolase | [IPI00514090](http://shh-b7892952567/mascot/cgi/protein_view.pl?file=../data/20140403/F001631.dat&hit=IPI00514090&px=1&ave_thresh=38&_sigthreshold=0.05&_server_mudpit_switch=0.001) | 61236 | 42 | 4 | 0.06 |
| 22 | C4A Complement C4-A | [IPI00032258](http://shh-b7892952567/mascot/cgi/protein_view.pl?file=../data/20140403/F001629.dat&hit=IPI00032258&px=1&ave_thresh=38&_sigthreshold=0.05&_server_mudpit_switch=0.001) | 194247 | 41 | 2 | 0.02 |

**Supplementary datasheet B** Proteins identified by Mass Spectrometry in NCM2

| ID | Gene Description | Accession  Number | Mass | Ions score>38, *P*<0.05 | Queries matched | emPAI |
| --- | --- | --- | --- | --- | --- | --- |
| 1 | ALB Isoform 1 of Serum albumin precursor | IPI00745872 | 71317 | 5228 | 278 | 38 |
| 2 | ALB Putative uncharacterized protein ALB | [IPI00022434](http://shh-b7892952567/mascot/cgi/protein_view.pl?file=../data/20140403/F001630.dat&hit=IPI00022434&px=1&ave_thresh=38&_sigthreshold=0.05&_server_mudpit_switch=0.001) | 73881 | 5034 | 269 | 31 |
| 3 | LOC100294459 similar to immunoglobulin lambda-like polypeptide 1 | [IPI00936490](http://shh-b7892952567/mascot/cgi/protein_view.pl?file=../data/20140403/F001640.dat&hit=IPI00936490&px=1&ave_thresh=38&_sigthreshold=0.05&_server_mudpit_switch=0.001) | 53960 | 713 | 32 | 0 |
| 4 | IGHG2 Putative uncharacterized protein DKFZp686C15213 | IPI00426051 | 51864 | 547 | 30 | 1 |
| 5 | TF Serotransferrin | [IPI00022463](http://shh-b7892952567/mascot/cgi/protein_view.pl?file=../data/20140403/F001630.dat&hit=IPI00022463&px=1&ave_thresh=38&_sigthreshold=0.05&_server_mudpit_switch=0.001) | 79280 | 502 | 19 | 1 |
| 6 | SERPINA1 Isoform 1 of Alpha-1-antitrypsin | [IPI00553177](http://shh-b7892952567/mascot/cgi/protein_view.pl?file=../data/20140403/F001640.dat&hit=IPI00553177&px=1&ave_thresh=38&_sigthreshold=0.05&_server_mudpit_switch=0.001) | 46878 | 256 | 26 | 1 |
| 7 | ITIH4 Isoform 2 of Inter-alpha-trypsin inhibitor heavy chain H4 | IPI00218192 | 101520 | 178 | 6 | 0 |
| 8 | IGHV4-31 Putative uncharacterized protein DKFZp686N02209 | [IPI00384938](http://shh-b7892952567/mascot/cgi/protein_view.pl?file=../data/20140403/F001640.dat&hit=IPI00384938&px=1&ave_thresh=38&_sigthreshold=0.05&_server_mudpit_switch=0.001) | 53503 | 145 | 15 | 0 |
| 9 | HBA1;HBA2 Hemoglobin subunit alpha | IPI00410714 | 15305 | 143 | 16 | 2 |
| 10 | LTA4H Isoform 2 of Leukotriene A-4 hydrolase | [IPI00514090](http://shh-b7892952567/mascot/cgi/protein_view.pl?file=../data/20140403/F001630.dat&hit=IPI00514090&px=1&ave_thresh=38&_sigthreshold=0.05&_server_mudpit_switch=0.001) | 61236 | 142 | 5 | 0 |
| 11 | IGHG2 Putative uncharacterized protein DKFZp686I04196 (Fragment) | [IPI00399007](http://shh-b7892952567/mascot/cgi/protein_view.pl?file=../data/20140403/F001640.dat&hit=IPI00399007&px=1&ave_thresh=38&_sigthreshold=0.05&_server_mudpit_switch=0.001) | 46716 | 129 | 8 | 0 |
| 12 | A2M Alpha-2-macroglobulin | [IPI00478003](http://shh-b7892952567/mascot/cgi/protein_view.pl?file=../data/20140403/F001640.dat&hit=IPI00478003&px=1&ave_thresh=38&_sigthreshold=0.05&_server_mudpit_switch=0.001) | 164600 | 117 | 5 | 0 |
| 13 | SERPINA3 Isoform 1 of Alpha-1-antichymotrypsin | [IPI00847635](http://shh-b7892952567/mascot/cgi/protein_view.pl?file=../data/20140403/F001630.dat&hit=IPI00847635&px=1&ave_thresh=38&_sigthreshold=0.05&_server_mudpit_switch=0.001) | 47792 | 111 | 3 | 0 |
| 14 | GC Vitamin D-binding protein | [IPI00555812](http://shh-b7892952567/mascot/cgi/protein_view.pl?file=../data/20140403/F001630.dat&hit=IPI00555812&px=1&ave_thresh=38&_sigthreshold=0.05&_server_mudpit_switch=0.001) | 54526 | 102 | 2 | 0 |
| 15 | PKM2 Isoform M1 of Pyruvate kinase isozymes M1/M2 | [IPI00220644](http://shh-b7892952567/mascot/cgi/protein_view.pl?file=../data/20140403/F001630.dat&hit=IPI00220644&px=1&ave_thresh=38&_sigthreshold=0.05&_server_mudpit_switch=0.001) | 58538 | 99 | 1 | 0 |
| 16 | Putative uncharacterized protein | [IPI00550731](http://shh-b7892952567/mascot/cgi/protein_view.pl?file=../data/20140403/F001640.dat&hit=IPI00550731&px=1&ave_thresh=38&_sigthreshold=0.05&_server_mudpit_switch=0.001) | 26503 | 97 | 2 | 0 |
| 17 | IGHG4 Putative uncharacterized protein IGHG4 (Fragment) | [IPI00830132](http://shh-b7892952567/mascot/cgi/protein_view.pl?file=../data/20140403/F001640.dat&hit=IPI00830132&px=1&ave_thresh=38&_sigthreshold=0.05&_server_mudpit_switch=0.001) | 43856 | 93 | 2 | 0 |
| 18 | YWHAE 14-3-3 protein epsilon | [IPI00000816](http://shh-b7892952567/mascot/cgi/protein_view.pl?file=../data/20140403/F001630.dat&hit=IPI00000816&px=1&ave_thresh=38&_sigthreshold=0.05&_server_mudpit_switch=0.001) | 29326 | 87 | 2 | 0 |
| 19 | C4A Complement C4-A | [IPI00032258](http://shh-b7892952567/mascot/cgi/protein_view.pl?file=../data/20140403/F001630.dat&hit=IPI00032258&px=1&ave_thresh=38&_sigthreshold=0.05&_server_mudpit_switch=0.001) | 194247 | 84 | 2 | 0 |
| 20 | A1BG Alpha-1B-glycoprotein | [IPI00022895](http://shh-b7892952567/mascot/cgi/protein_view.pl?file=../data/20140403/F001630.dat&hit=IPI00022895&px=1&ave_thresh=38&_sigthreshold=0.05&_server_mudpit_switch=0.001) | 54809 | 79 | 2 | 0 |
| 21 | CAP1 Isoform 1 of Adenylyl cyclase-associated protein 1 | [IPI00008274](http://shh-b7892952567/mascot/cgi/protein_view.pl?file=../data/20140403/F001630.dat&hit=IPI00008274&px=1&ave_thresh=38&_sigthreshold=0.05&_server_mudpit_switch=0.001) | 52222 | 75 | 4 | 0 |
| 22 | GPI Glucose-6-phosphate isomerase | [IPI00027497](http://shh-b7892952567/mascot/cgi/protein_view.pl?file=../data/20140403/F001630.dat&hit=IPI00027497&px=1&ave_thresh=38&_sigthreshold=0.05&_server_mudpit_switch=0.001) | 63335 | 73 | 2 | 0 |
| 23 | ENO1 Isoform alpha-enolase of Alpha-enolase | [IPI00465248](http://shh-b7892952567/mascot/cgi/protein_view.pl?file=../data/20140403/F001630.dat&hit=IPI00465248&px=1&ave_thresh=38&_sigthreshold=0.05&_server_mudpit_switch=0.001) | 47481 | 63 | 5 | 0 |
| 24 | AGT Angiotensinogen | [IPI00032220](http://shh-b7892952567/mascot/cgi/protein_view.pl?file=../data/20140403/F001630.dat&hit=IPI00032220&px=1&ave_thresh=38&_sigthreshold=0.05&_server_mudpit_switch=0.001) | 53406 | 60 | 1 | 0 |
| 25 | PRED58 protein (Fragment) | [REV_IPI00022050](http://shh-b7892952567/mascot/cgi/protein_view.pl?file=../data/20140403/F001630.dat&hit=REV_IPI00022050&px=1&ave_thresh=38&_sigthreshold=0.05&_server_mudpit_switch=0.001) |  | 55 | 11 | 0 |
| 26 | highly similar to Alpha-2-HS-glycoprotein | [IPI00022431](http://shh-b7892952567/mascot/cgi/protein_view.pl?file=../data/20140403/F001640.dat&hit=IPI00022431&px=1&ave_thresh=38&_sigthreshold=0.05&_server_mudpit_switch=0.001) | 47567 | 50 | 1 | 0 |
| 27 | IGHA1 SNC66 protein | [IPI00383164](http://shh-b7892952567/mascot/cgi/protein_view.pl?file=../data/20140403/F001640.dat&hit=IPI00383164&px=1&ave_thresh=38&_sigthreshold=0.05&_server_mudpit_switch=0.001) | 54601 | 49 | 2 | 0 |
| 28 | ARF4 ADP-ribosylation factor 4 | [IPI00215918](http://shh-b7892952567/mascot/cgi/protein_view.pl?file=../data/20140403/F001630.dat&hit=IPI00215918&px=1&ave_thresh=38&_sigthreshold=0.05&_server_mudpit_switch=0.001) | 20612 | 44 | 9 | 0 |
| 29 | IGHG1 | [IPI00892671](http://shh-b7892952567/mascot/cgi/protein_view.pl?file=../data/20140403/F001630.dat&hit=IPI00892671&px=1&ave_thresh=38&_sigthreshold=0.05&_server_mudpit_switch=0.001) | 32476 | 44 | 9 | 0 |
| 30 | C3 Complement C3 (Fragment) | [IPI00783987](http://shh-b7892952567/mascot/cgi/protein_view.pl?file=../data/20140403/F001630.dat&hit=IPI00783987&px=1&ave_thresh=38&_sigthreshold=0.05&_server_mudpit_switch=0.001) | 188569 | 44 | 3 | 0 |
| 31 | IGHV4-31 Putative uncharacterized protein | [IPI00384938](http://shh-b7892952567/mascot/cgi/protein_view.pl?file=../data/20140403/F001630.dat&hit=IPI00384938&px=1&ave_thresh=38&_sigthreshold=0.05&_server_mudpit_switch=0.001) | 53503 | 43 | 9 | 0 |
| 32 | IGHV4-31 Putative uncharacterized protein | [IPI00930124](http://shh-b7892952567/mascot/cgi/protein_view.pl?file=../data/20140403/F001630.dat&hit=IPI00930124&px=1&ave_thresh=38&_sigthreshold=0.05&_server_mudpit_switch=0.001) | 52829 | 43 | 9 | 0 |
| 33 | F13A1 Coagulation factor XIII A chain | [IPI00297550](http://shh-b7892952567/mascot/cgi/protein_view.pl?file=../data/20140403/F001630.dat&hit=IPI00297550&px=1&ave_thresh=38&_sigthreshold=0.05&_server_mudpit_switch=0.001) | 83728 | 42 | 2 | 0 |

**Supplementary datasheet C** Proteins identified by Mass Spectrometry in NCM3

| ID | Gene Description | Accession  Number | Mass | Ions score>38, *P*<0.05 | Queries matched | emPAI |
| --- | --- | --- | --- | --- | --- | --- |
| 1 | ALB Putative uncharacterized protein ALB | [IPI00022434](http://shh-b7892952567/mascot/cgi/protein_view.pl?file=../data/20140403/F001634.dat&hit=IPI00022434&px=1&ave_thresh=38&_sigthreshold=0.05&_server_mudpit_switch=0.001) | 73881 | 5193 | 287 | 24 |
| 2 | IGHG2 Putative uncharacterized protein DKFZp686C15213 | [IPI00426051](http://shh-b7892952567/mascot/cgi/protein_view.pl?file=../data/20140403/F001634.dat&hit=IPI00426051&px=1&ave_thresh=38&_sigthreshold=0.05&_server_mudpit_switch=0.001) | 51864 | 828 | 41 | 1 |
| 3 | TF Serotransferrin | [IPI00022463](http://shh-b7892952567/mascot/cgi/protein_view.pl?file=../data/20140403/F001634.dat&hit=IPI00022463&px=1&ave_thresh=38&_sigthreshold=0.05&_server_mudpit_switch=0.001) | 79280 | 668 | 34 | 1 |
| 4 | HBA1;HBA2 Hemoglobin subunit alpha | [IPI00410714](http://shh-b7892952567/mascot/cgi/protein_view.pl?file=../data/20140403/F001634.dat&hit=IPI00410714&px=1&ave_thresh=38&_sigthreshold=0.05&_server_mudpit_switch=0.001) | 15305 | 451 | 26 | 18 |
| 5 | HBB Hemoglobin subunit beta | [IPI00654755](http://shh-b7892952567/mascot/cgi/protein_view.pl?file=../data/20140403/F001634.dat&hit=IPI00654755&px=1&ave_thresh=38&_sigthreshold=0.05&_server_mudpit_switch=0.001) | 16102 | 420 | 20 | 12 |
| 6 | Putative uncharacterized protein | [IPI00550731](http://shh-b7892952567/mascot/cgi/protein_view.pl?file=../data/20140403/F001634.dat&hit=IPI00550731&px=1&ave_thresh=38&_sigthreshold=0.05&_server_mudpit_switch=0.001) | 26503 | 405 | 15 | 2 |
| 7 | LTA4H cDNA FLJ51009, highly similar to Leukotriene A-4 hydrolase | [IPI00793812](http://shh-b7892952567/mascot/cgi/protein_view.pl?file=../data/20140403/F001634.dat&hit=IPI00793812&px=1&ave_thresh=38&_sigthreshold=0.05&_server_mudpit_switch=0.001) | 57590 | 251 | 9 | 0 |
| 8 | IGHG1 44 kDa protein | [IPI00448925](http://shh-b7892952567/mascot/cgi/protein_view.pl?file=../data/20140403/F001634.dat&hit=IPI00448925&px=1&ave_thresh=38&_sigthreshold=0.05&_server_mudpit_switch=0.001) | 44511 | 220 | 29 | 1 |
| 9 | IGHG4 Putative uncharacterized protein IGHG4 (Fragment) | [IPI00830132](http://shh-b7892952567/mascot/cgi/protein_view.pl?file=../data/20140403/F001634.dat&hit=IPI00830132&px=1&ave_thresh=38&_sigthreshold=0.05&_server_mudpit_switch=0.001) | 43856 | 219 | 7 | 0 |
| 10 | IGHV4-31 Putative uncharacterized protein DKFZp686C11235 | [IPI00930124](http://shh-b7892952567/mascot/cgi/protein_view.pl?file=../data/20140403/F001634.dat&hit=IPI00930124&px=1&ave_thresh=38&_sigthreshold=0.05&_server_mudpit_switch=0.001) | 52829 | 200 | 26 | 1 |
| 11 | SERPINA1 Isoform 2 of Alpha-1-antitrypsin | [IPI00790784](http://shh-b7892952567/mascot/cgi/protein_view.pl?file=../data/20140403/F001634.dat&hit=IPI00790784&px=1&ave_thresh=38&_sigthreshold=0.05&_server_mudpit_switch=0.001) | 40409 | 187 | 27 | 1 |
| 12 | SERPINA1 Isoform 1 of Alpha-1-antitrypsin | [IPI00553177](http://shh-b7892952567/mascot/cgi/protein_view.pl?file=../data/20140403/F001634.dat&hit=IPI00553177&px=1&ave_thresh=38&_sigthreshold=0.05&_server_mudpit_switch=0.001) | 46878 | 186 | 28 | 1 |
| 13 | HP Haptoglobin | [IPI00641737](http://shh-b7892952567/mascot/cgi/protein_view.pl?file=../data/20140403/F001634.dat&hit=IPI00641737&px=1&ave_thresh=38&_sigthreshold=0.05&_server_mudpit_switch=0.001) | 47378 | 154 | 9 | 0 |
| 14 | ITIH4 Isoform 2 of Inter-alpha-trypsin inhibitor heavy chain H4 | [IPI00218192](http://shh-b7892952567/mascot/cgi/protein_view.pl?file=../data/20140403/F001634.dat&hit=IPI00218192&px=1&ave_thresh=38&_sigthreshold=0.05&_server_mudpit_switch=0.001) | 101520 | 140 | 3 | 0 |
| 15 | FGB Fibrinogen beta chain | [IPI00298497](http://shh-b7892952567/mascot/cgi/protein_view.pl?file=../data/20140403/F001634.dat&hit=IPI00298497&px=1&ave_thresh=38&_sigthreshold=0.05&_server_mudpit_switch=0.001) | 56577 | 116 | 7 | 0 |
| 16 | IGLC1;IGLV2-110 | [IPI00154742](http://shh-b7892952567/mascot/cgi/protein_view.pl?file=../data/20140403/F001634.dat&hit=IPI00154742&px=1&ave_thresh=38&_sigthreshold=0.05&_server_mudpit_switch=0.001) | 25119 | 113 | 8 | 0 |
| 17 | HBD;HBB Hemoglobin subunit delta | [IPI00473011](http://shh-b7892952567/mascot/cgi/protein_view.pl?file=../data/20140403/F001634.dat&hit=IPI00473011&px=1&ave_thresh=38&_sigthreshold=0.05&_server_mudpit_switch=0.001) | 16159 | 111 | 7 | 1 |
| 18 | PGM1 Isoform 2 of Phosphoglucomutase-1 | [IPI00217872](http://shh-b7892952567/mascot/cgi/protein_view.pl?file=../data/20140403/F001634.dat&hit=IPI00217872&px=1&ave_thresh=38&_sigthreshold=0.05&_server_mudpit_switch=0.001) | 64092 | 79 | 2 | 0 |
| 19 | C5 Complement C5 | [IPI00032291](http://shh-b7892952567/mascot/cgi/protein_view.pl?file=../data/20140403/F001634.dat&hit=IPI00032291&px=1&ave_thresh=38&_sigthreshold=0.05&_server_mudpit_switch=0.001) | 189897 | 69 | 2 | 0 |
| 20 | FGG Isoform Gamma-B of Fibrinogen gamma chain | [IPI00021891](http://shh-b7892952567/mascot/cgi/protein_view.pl?file=../data/20140403/F001634.dat&hit=IPI00021891&px=1&ave_thresh=38&_sigthreshold=0.05&_server_mudpit_switch=0.001) | 52106 | 62 | 4 | 0 |
| 21 | HPX Hemopexin | [IPI00022488](http://shh-b7892952567/mascot/cgi/protein_view.pl?file=../data/20140403/F001634.dat&hit=IPI00022488&px=1&ave_thresh=38&_sigthreshold=0.05&_server_mudpit_switch=0.001) | 52385 | 58 | 3 | 0 |
| 22 | LUM Lumican | [IPI00020986](http://shh-b7892952567/mascot/cgi/protein_view.pl?file=../data/20140403/F001634.dat&hit=IPI00020986&px=1&ave_thresh=38&_sigthreshold=0.05&_server_mudpit_switch=0.001) | 38747 | 55 | 1 | 0 |
| 23 | C9 Complement component C9 | [IPI00022395](http://shh-b7892952567/mascot/cgi/protein_view.pl?file=../data/20140403/F001634.dat&hit=IPI00022395&px=1&ave_thresh=38&_sigthreshold=0.05&_server_mudpit_switch=0.001) | 64615 | 53 | 1 | 0 |
| 24 | IGHG3 FLJ00385 protein (Fragment | [IPI00168728](http://shh-b7892952567/mascot/cgi/protein_view.pl?file=../data/20140403/F001634.dat&hit=IPI00168728&px=1&ave_thresh=38&_sigthreshold=0.05&_server_mudpit_switch=0.001) | 57272 | 52 | 6 | 0 |
| 25 | C3 Complement C3 (Fragment) | [IPI00783987](http://shh-b7892952567/mascot/cgi/protein_view.pl?file=../data/20140403/F001634.dat&hit=IPI00783987&px=1&ave_thresh=38&_sigthreshold=0.05&_server_mudpit_switch=0.001) | 188569 | 49 | 5 | 0 |
| 26 | IGHA1 SNC66 protein | [IPI00383164](http://shh-b7892952567/mascot/cgi/protein_view.pl?file=../data/20140403/F001634.dat&hit=IPI00383164&px=1&ave_thresh=38&_sigthreshold=0.05&_server_mudpit_switch=0.001) | 54601 | 43 | 3 | 0 |
| 27 | CP Ceruloplasmin | [IPI00017601](http://shh-b7892952567/mascot/cgi/protein_view.pl?file=../data/20140403/F001634.dat&hit=IPI00017601&px=1&ave_thresh=38&_sigthreshold=0.05&_server_mudpit_switch=0.001) | 122983 | 41 | 8 | 0 |
| 28 | HBG1;HBG2 Hemoglobin subunit gamma-1 | [IPI00220706](http://shh-b7892952567/mascot/cgi/protein_view.pl?file=../data/20140403/F001634.dat&hit=IPI00220706&px=1&ave_thresh=38&_sigthreshold=0.05&_server_mudpit_switch=0.001) | 16187 | 40 | 2 | 0 |
| 29 | C4A Complement C4-A | [IPI00032258](http://shh-b7892952567/mascot/cgi/protein_view.pl?file=../data/20140403/F001634.dat&hit=IPI00032258&px=1&ave_thresh=38&_sigthreshold=0.05&_server_mudpit_switch=0.001) | 194247 | 39 | 3 | 0 |

**Supplementary datasheet D** Proteins identified by Mass Spectrometry in NCM4

| ID | Gene Description | Accession Number | Mass | Ions score>38, *P*<0.05 | Queries matched | emPAI |
| --- | --- | --- | --- | --- | --- | --- |
| 1 | ALB Putative uncharacterized protein ALB | IPI00022434 | 73881 | 5019 | 263 | 22 |
| 2 | IGHG2 Putative uncharacterized protein DKFZp686C15213 | [IPI00426051](http://shh-b7892952567/mascot/cgi/protein_view.pl?file=../data/20140403/F001633.dat&hit=IPI00426051&px=1&ave_thresh=38&_sigthreshold=0.05&_server_mudpit_switch=0.001) | 51864 | 950 | 53 | 1 |
| 3 | TF Serotransferrin | [IPI00022463](http://shh-b7892952567/mascot/cgi/protein_view.pl?file=../data/20140403/F001633.dat&hit=IPI00022463&px=1&ave_thresh=38&_sigthreshold=0.05&_server_mudpit_switch=0.001) | 79280 | 841 | 36 | 1 |
| 4 | IGHG1 44 kDa protein | [IPI00448925](http://shh-b7892952567/mascot/cgi/protein_view.pl?file=../data/20140403/F001633.dat&hit=IPI00448925&px=1&ave_thresh=38&_sigthreshold=0.05&_server_mudpit_switch=0.001) | 44511 | 476 | 36 | 2 |
| 5 | IGHV4-31 Putative uncharacterized protein DKFZp686C11235 | [IPI00930124](http://shh-b7892952567/mascot/cgi/protein_view.pl?file=../data/20140403/F001633.dat&hit=IPI00930124&px=1&ave_thresh=38&_sigthreshold=0.05&_server_mudpit_switch=0.001) | 52829 | 473 | 36 | 1 |
| 6 | IGHG2 Putative uncharacterized protein DKFZp686I04196 (Fragment) | [IPI00399007](http://shh-b7892952567/mascot/cgi/protein_view.pl?file=../data/20140403/F001633.dat&hit=IPI00399007&px=1&ave_thresh=38&_sigthreshold=0.05&_server_mudpit_switch=0.001) | 46716 | 342 | 23 | 1 |
| 7 | Putative uncharacterized protein | [IPI00550731](http://shh-b7892952567/mascot/cgi/protein_view.pl?file=../data/20140403/F001633.dat&hit=IPI00550731&px=1&ave_thresh=38&_sigthreshold=0.05&_server_mudpit_switch=0.001) | 26503 | 219 | 4 | 1 |
| 8 | ITIH4 Isoform 2 of Inter-alpha-trypsin inhibitor heavy chain H4 | [IPI00218192](http://shh-b7892952567/mascot/cgi/protein_view.pl?file=../data/20140403/F001633.dat&hit=IPI00218192&px=1&ave_thresh=38&_sigthreshold=0.05&_server_mudpit_switch=0.001) | 101520 | 207 | 6 | 0 |
| 9 | IGHA1 Putative uncharacterized protein DKFZp686G21220 (Fragment) | [IPI00423460](http://shh-b7892952567/mascot/cgi/protein_view.pl?file=../data/20140403/F001633.dat&hit=IPI00423460&px=1&ave_thresh=38&_sigthreshold=0.05&_server_mudpit_switch=0.001) | 55323 | 206 | 6 | 0 |
| 10 | IGHA1 SNC66 protein | [IPI00383164](http://shh-b7892952567/mascot/cgi/protein_view.pl?file=../data/20140403/F001633.dat&hit=IPI00383164&px=1&ave_thresh=38&_sigthreshold=0.05&_server_mudpit_switch=0.001) | 54601 | 204 | 7 | 0 |
| 11 | C3 Complement C3 (Fragment) | [IPI00783987](http://shh-b7892952567/mascot/cgi/protein_view.pl?file=../data/20140403/F001633.dat&hit=IPI00783987&px=1&ave_thresh=38&_sigthreshold=0.05&_server_mudpit_switch=0.001) | 188569 | 170 | 12 | 0 |
| 12 | SERPINA1 Isoform 1 of Alpha-1-antitrypsin | [IPI00553177](http://shh-b7892952567/mascot/cgi/protein_view.pl?file=../data/20140403/F001633.dat&hit=IPI00553177&px=1&ave_thresh=38&_sigthreshold=0.05&_server_mudpit_switch=0.001) | 46878 | 158 | 6 | 1 |
| 13 | IGHG4 Putative uncharacterized protein IGHG4 (Fragment) | [IPI00830132](http://shh-b7892952567/mascot/cgi/protein_view.pl?file=../data/20140403/F001633.dat&hit=IPI00830132&px=1&ave_thresh=38&_sigthreshold=0.05&_server_mudpit_switch=0.001) | 43856 | 154 | 5 | 0 |
| 14 | IGHG4 Putative uncharacterized protein DKFZp686M24218 | [IPI00930442](http://shh-b7892952567/mascot/cgi/protein_view.pl?file=../data/20140403/F001633.dat&hit=IPI00930442&px=1&ave_thresh=38&_sigthreshold=0.05&_server_mudpit_switch=0.001) | 53071 | 147 | 4 | 0 |
| 15 | HSP90AB1 Heat shock protein HSP 90-beta | [IPI00414676](http://shh-b7892952567/mascot/cgi/protein_view.pl?file=../data/20140403/F001644.dat&hit=IPI00414676&px=1&ave_thresh=38&_sigthreshold=0.05&_server_mudpit_switch=0.001) | 83554 | 126 | 2 | 0 |
| 16 | HPX Hemopexin | [IPI00022488](http://shh-b7892952567/mascot/cgi/protein_view.pl?file=../data/20140403/F001633.dat&hit=IPI00022488&px=1&ave_thresh=38&_sigthreshold=0.05&_server_mudpit_switch=0.001) | 52385 | 123 | 5 | 0 |
| 17 | highly similar to Complement factor B | [IPI00019591](http://shh-b7892952567/mascot/cgi/protein_view.pl?file=../data/20140403/F001633.dat&hit=IPI00019591&px=1&ave_thresh=38&_sigthreshold=0.05&_server_mudpit_switch=0.001) | 143191 | 117 | 7 | 0 |
| 18 | IGLC1 | [IPI00154742](http://shh-b7892952567/mascot/cgi/protein_view.pl?file=../data/20140403/F001633.dat&hit=IPI00154742&px=1&ave_thresh=38&_sigthreshold=0.05&_server_mudpit_switch=0.001) | 25119 | 115 | 7 | 0 |
| 19 | LUM Lumican | [IPI00020986](http://shh-b7892952567/mascot/cgi/protein_view.pl?file=../data/20140403/F001633.dat&hit=IPI00020986&px=1&ave_thresh=38&_sigthreshold=0.05&_server_mudpit_switch=0.001) | 38747 | 102 | 2 | 0 |
| 20 | LCP1 Plastin-2 | [IPI00010471](http://shh-b7892952567/mascot/cgi/protein_view.pl?file=../data/20140403/F001633.dat&hit=IPI00010471&px=1&ave_thresh=38&_sigthreshold=0.05&_server_mudpit_switch=0.001) | 70815 | 87 | 2 | 0 |
| 21 | GPI Glucose-6-phosphate isomerase | [IPI00027497](http://shh-b7892952567/mascot/cgi/protein_view.pl?file=../data/20140403/F001633.dat&hit=IPI00027497&px=1&ave_thresh=38&_sigthreshold=0.05&_server_mudpit_switch=0.001) | 63335 | 82 | 6 | 0 |
| 22 | PGM1 Isoform 2 of Phosphoglucomutase-1 | [IPI00217872](http://shh-b7892952567/mascot/cgi/protein_view.pl?file=../data/20140403/F001633.dat&hit=IPI00217872&px=1&ave_thresh=38&_sigthreshold=0.05&_server_mudpit_switch=0.001) | 64092 | 78 | 1 | 0 |
| 23 | C9 Complement component C9 | [IPI00022395](http://shh-b7892952567/mascot/cgi/protein_view.pl?file=../data/20140403/F001633.dat&hit=IPI00022395&px=1&ave_thresh=38&_sigthreshold=0.05&_server_mudpit_switch=0.001) | 64615 | 78 | 1 | 0 |
| 24 | PKM2 Isoform M1 of Pyruvate kinase isozymes M1/M2 | [IPI00220644](http://shh-b7892952567/mascot/cgi/protein_view.pl?file=../data/20140403/F001633.dat&hit=IPI00220644&px=1&ave_thresh=38&_sigthreshold=0.05&_server_mudpit_switch=0.001) | 58538 | 74 | 2 | 0 |
| 25 | C4A Complement C4-A | [IPI00032258](http://shh-b7892952567/mascot/cgi/protein_view.pl?file=../data/20140403/F001633.dat&hit=IPI00032258&px=1&ave_thresh=38&_sigthreshold=0.05&_server_mudpit_switch=0.001) | 194247 | 69 | 2 | 0 |
| 26 | A2M Alpha-2-macroglobulin | [IPI00478003](http://shh-b7892952567/mascot/cgi/protein_view.pl?file=../data/20140403/F001644.dat&hit=IPI00478003&px=1&ave_thresh=38&_sigthreshold=0.05&_server_mudpit_switch=0.001) | 164600 | 60 | 3 | 0 |
| 27 | PRED58 protein (Fragment) | [REV IPI00022050](http://shh-b7892952567/mascot/cgi/protein_view.pl?file=../data/20140403/F001633.dat&hit=REV_IPI00022050&px=1&ave_thresh=38&_sigthreshold=0.05&_server_mudpit_switch=0.001) |  | 50 | 9 |  |
| 28 | SERPINC1 Antithrombin-III | [IPI00032179](http://shh-b7892952567/mascot/cgi/protein_view.pl?file=../data/20140403/F001644.dat&hit=IPI00032179&px=1&ave_thresh=38&_sigthreshold=0.05&_server_mudpit_switch=0.001) | 53025 | 49 | 2 | 0 |
| 29 | SDF2L1 Dihydropyrimidinase-like 2 | [IPI00106642](http://shh-b7892952567/mascot/cgi/protein_view.pl?file=../data/20140403/F001633.dat&hit=IPI00106642&px=1&ave_thresh=38&_sigthreshold=0.05&_server_mudpit_switch=0.001) | 67545 | 42 | 1 | 0 |
| 30 | CP Ceruloplasmin | [IPI00017601](http://shh-b7892952567/mascot/cgi/protein_view.pl?file=../data/20140403/F001633.dat&hit=IPI00017601&px=1&ave_thresh=38&_sigthreshold=0.05&_server_mudpit_switch=0.001) | 122983 | 42 | 3 | 0 |
| 31 | VCL Isoform 1 of Vinculin | [IPI00291175](http://shh-b7892952567/mascot/cgi/protein_view.pl?file=../data/20140403/F001633.dat&hit=IPI00291175&px=1&ave_thresh=38&_sigthreshold=0.05&_server_mudpit_switch=0.001) | 117220 | 41 | 1 | 0 |
| 32 | HBA1;HBA2 Hemoglobin subunit alpha | [IPI00410714](http://shh-b7892952567/mascot/cgi/protein_view.pl?file=../data/20140403/F001644.dat&hit=IPI00410714&px=1&ave_thresh=38&_sigthreshold=0.05&_server_mudpit_switch=0.001) | 15305 | 40 | 20 | 0 |
| 33 | ITIH4 Isoform 2 of Inter-alpha-trypsin inhibitor heavy chain H4 | [IPI00218192](http://shh-b7892952567/mascot/cgi/protein_view.pl?file=../data/20140403/F001644.dat&hit=IPI00218192&px=1&ave_thresh=38&_sigthreshold=0.05&_server_mudpit_switch=0.001) | 101520 | 39 | 1 | 0 |
